# A pilot study of the feasibility and preliminary outcomes of sequential TF-CBT and EMDR online group therapy for adult women with histories of childhood sexual abuse

**DOI:** 10.3389/fpsyg.2026.1872104

**Published:** 2026-07-15

**Authors:** Milagros Molero-Zafra, María Jesús Hernández-Jiménez, Olga Fernández-García, Marián Pérez-Marín

**Affiliations:** 1 Faculty of Psychology, University of Valencia, Valencia, Spain; 2 Mental Health Unit (USM) Sedaví, Dr. Peset Hospital Health Department, Valencia, Spain

**Keywords:** childhood sexual abuse, complex trauma, dissociation, EMDR G-TEP, online intervention, pilot study, TF-CBT

## Abstract

**Background:**

CSA is associated with post-traumatic stress disorder (PTSD), dissociation, emotion-regulation difficulties and psychopathology. Trauma-focused cognitive behavioral therapy (TF-CBT) and eye movement desensitization and reprocessing (EMDR) are evidence-based interventions, but their sequential delivery in online groups for women with CSA-related complex trauma remains underexplored.

**Objective:**

To examine feasibility and preliminary clinical outcomes of a 16-session online group intervention combining TF-CBT and EMDR Group Traumatic Episode Protocol (G-TEP), and to explore differences by sequence (EMDR → CBT vs. CBT → EMDR).

**Methods:**

Of 31 eligible participants, nine adult women (Mean age = 39.42 years, SD = 10.23) completed both phases and assessment (EMDR → CBT: *n* = 5; CBT → EMDR: *n* = 4). Assessments at baseline (T1), T2, and T3 included PTSD (EGS-R), dissociation (DES), psychopathology (SCL-90-R), emotion regulation (DERS), self-esteem (RSES), life satisfaction (SWLS), and treatment satisfaction (CRES-4). Non-parametric tests and effect sizes were used. An illustrative case was included.

**Results:**

The intervention showed descriptive improvements across domains, including PTSD symptoms, dissociation, and psychopathology, with improved self-esteem and life satisfaction. However, most statistical tests were non-significant, consistent with the exploratory nature of the small sample. Effect sizes indicated variability across sequences: EMDR → CBT showed larger effects in phobic anxiety and self-esteem, whereas CBT → EMDR showed larger effects in dissociation. Findings indicate exploratory sequence-related patterns rather than definitive treatment effects.

**Conclusion:**

Sequential online delivery of TF-CBT and EMDR G-TEP appears feasible among completers, although substantial attrition limits feasibility. Findings are preliminary and require confirmation in future controlled studies.

## 1 Introduction

Childhood sexual abuse (CSA) is a severe form of early interpersonal trauma associated with persistent psychological consequences, including post-traumatic stress disorder (PTSD), dissociation, emotion-regulation difficulties, impaired self-concept, and interpersonal problems (Pereda and Abad, 2013; Teicher and Samson, 2016). These symptoms often continue into adulthood and are frequently accompanied by comorbidity, chronicity, functional impairment, and difficulties sustaining trauma-focused treatment.

Trauma-focused cognitive behavioral therapy (TF-CBT) and eye movement desensitization and reprocessing (EMDR) are recommended first-line treatments for trauma-related disorders (World Health Organization, 2013; National Institute for Health and Care Excellence, 2018). TF-CBT emphasizes psychoeducation, emotion regulation, cognitive restructuring, and trauma narrative work (Cohen et al., 2017), whereas EMDR aims to reprocess maladaptively stored traumatic memories through the Adaptive Information Processing model (Shapiro, 2018).

Meta-analytic evidence supports trauma-focused treatments for PTSD, with moderate to large symptom reductions and no evidence of overall clinical worsening despite possible transient symptom activation during treatment (Hoppen et al., 2023; Phillips et al., 2024; Purnell et al., 2024). However, CSA-related complex trauma may require attention to both stabilization and trauma processing. In women with CSA histories, previous work suggests partially distinct change patterns: TF-CBT may support emotion regulation and cognitive organization, whereas EMDR may be especially relevant for dissociation and trauma integration (Molero-Zafra et al., 2024).

Online and group formats may increase access to specialized trauma care for CSA survivors, who often face stigma, avoidance, and limited availability of individual treatment. Group interventions may also provide normalization, interpersonal learning, and social support (Yalom and Leszcz, 2020). Within EMDR, the Group Traumatic Episode Protocol (G-TEP) offers a structured method for trauma processing in groups while preserving individualized work (Jarero and Artigas, 2012; Lehnung et al., 2017; Shapiro, 2023).

However, evidence remains limited regarding sequential combinations of trauma-focused approaches, particularly in complex trauma populations with dissociative symptoms. Clinicians commonly combine stabilization and trauma-processing interventions, yet empirical guidance on optimal sequencing is scarce. Determining whether order matters is clinically relevant because a sequence that improves tolerability or targets dissociation more effectively could help refine treatment planning for survivors with high symptom burden. This question is especially important in services where group or online formats are used to increase access, because treatment length and sequencing have direct implications for adherence, therapist workload, and participant safety.

This pilot study evaluated the feasibility and preliminary clinical effects of a sequential online group intervention combining TF-CBT and EMDR G-TEP in adult women with CSA-related complex trauma. It also explored whether EMDR → CBT and CBT → EMDR were associated with different trajectories of clinical change. Given the small sample, the study was designed to generate feasibility data and effect-size signals rather than confirm efficacy. A detailed case study complemented quantitative findings.

## 2 Methods

### 2.1 Design

This pilot randomized exploratory study examined the feasibility and preliminary clinical effects of a sequential online group intervention combining TF-CBT and EMDR using the EMDR Group Traumatic Episode Protocol (G-TEP; Shapiro, 2023). The study used a two-arm sequence design with three repeated assessments, allowing preliminary examination of change after each treatment phase and after completion of the full protocol.

Participants were randomized to EMDR followed by TF-CBT (EMDR → CBT) or TF-CBT followed by EMDR (CBT → EMDR). Because the study was conceived as a pilot and the final sample was small, analyses were exploratory and were not powered to detect definitive between-group differences. Feasibility was evaluated through completion of the intervention and tolerance of the online group format.

An illustrative case study was also included.

### 2.2 Participants

Adult women with a history of CSA were recruited through specialized clinical services and trauma-focused support networks, particularly ACASI (Association against Childhood Sexual Abuse). Recruitment targeted women seeking trauma-focused psychological support and willing to participate in an online group intervention with repeated assessments.

Inclusion criteria were: (a) female gender, (b) age ≥ 18 years, and (c) history of CSA. Exclusion criteria were severe mental illness, active substance dependence, and concurrent trauma-focused psychological treatment.

For the present analyses, participants also had to complete both intervention phases and the three assessment points (T1: pretreatment; T2: after phase 1; T3: after phase 2).

Of 31 eligible women (M age = 39.42, SD = 10.23), most were Spanish, university educated, employed, and in stable relationships. CSA histories were typically early, chronic, repeated, and frequently intrafamilial, with low disclosure and reporting rates.

Nine women completed the full protocol and were included in the analyses (EMDR → CBT: *n* = 5; CBT → EMDR: *n* = 4).

The final sample reflects completers of a demanding 16-session, two-phase trauma-focused intervention. The attrition observed is consistent with the challenges reported in complex trauma and PTSD treatment studies, where avoidance, dissociation, symptom severity, and life instability can interfere with sustained engagement (Imel et al., 2013; Lewis et al., 2020). For this reason, the results are best understood as feasibility and preliminary clinical signals among participants able to complete the protocol.

### 2.3 Measures

Clinical outcomes were assessed at baseline (T1), after the first intervention phase (T2), and after the second intervention phase (T3). The three-assessment structure was selected to distinguish change associated with the first treatment phase from change after completion of the full sequential model. This was important because the clinical hypothesis concerned not only overall improvement, but also whether the order of stabilization-oriented and processing-oriented components might produce different symptom trajectories.

Standardized self-report instruments were used for PTSD symptoms (EGS-R; Echeburúa et al., 2016), dissociation (DES; Icaran et al., 1996), general psychopathology (SCL-90-R; Derogatis, 2002), self-esteem (RSES; Rosenberg, 1965), life satisfaction (SWLS; Diener et al., 1985), and treatment satisfaction (CRES-4; Feixas et al., 2012). Emotion regulation difficulties were assessed with the DERS (Gratz and Roemer, 2004). Sociodemographic and clinical data, including CSA characteristics, previous treatment, psychiatric and somatic history, and disclosure/reporting variables, were collected with an *ad hoc* registry.

Although DES scores are commonly reported as item means, summed scores were used in the present study (yielding a total score from 0 to 2800) to maximize sensitivity to within-subject change across repeated assessments. Findings should therefore be interpreted in relation to change over time rather than conventional clinical cut-off values.

All instruments have shown adequate psychometric properties and are commonly used in trauma-related research.

### 2.4 Procedure

The intervention was delivered online via Zoom. After baseline assessment, participants were randomly assigned to their initial treatment modality (TF-CBT or EMDR G-TEP). Upon completion of the first 8-week phase, participants crossed over to receive the alternate treatment for the second phase. Randomization for the initial allocation was performed using a computer-generated sequence by a researcher not involved in treatment delivery or assessment.

Assessments were completed individually through the secure LimeSurvey platform (University of Valencia) during synchronous group assessment sessions lasting approximately 2 h, with real-time instructions and support from the research team.

Two trauma-informed clinical psychologists led both assessment and treatment sessions to support safety and containment. Further details of the protocol are reported in Molero-Zafra et al. (2022).

Each participant received 16 weekly 60-mins sessions: eight TF-CBT sessions and eight EMDR G-TEP sessions. TF-CBT included psychoeducation, emotion-regulation strategies, cognitive restructuring, and trauma narrative components. EMDR G-TEP followed a structured group protocol based on the Adaptive Information Processing model and used individual worksheets within the group context to support trauma processing while preserving privacy.

Although one sequence began with EMDR, G-TEP includes stabilization procedures in each session, including grounding, breathing, safe-place work, and structured self-regulation (Shapiro, 2023). The online group format was paced by two clinicians, with attention to containment, participant readiness, and management of emotional activation during and after sessions.

Participants receiving psychotropic medication were allowed to continue their treatment provided the medication remained stable during the intervention period. Medication changes were not systematically monitored throughout the study.

### 2.5 Data analysis

Because of the limited sample size (*N* = 9), analyses focused on exploratory within-subject and between-group patterns of change. No parametric assumptions were made, and findings were interpreted primarily through descriptive change and effect sizes rather than statistical significance. This strategy was chosen to avoid overstating results while still extracting clinically useful information from a pilot sample.

Between-group differences at T1, T2, and T3 were examined with Mann-Whitney U tests. Effect sizes were calculated using rank-biserial correlation (r_rb), interpreted as small (0.10), medium (0.30), and large (0.50).

Within-subject changes across T1, T2, and T3 were examined with Friedman tests. Following methodological recommendations for non-parametric analyses (Tomczak and Tomczak, 2014), Kendall's *W* was used as the appropriate effect size measure (0.10 = small, 0.30 = moderate, ≥0.50 = large).

This approach allowed examination of cumulative change and possible sequence-related trajectories while avoiding overinterpretation of *p*-values in a small pilot sample.

### 2.6 Ethics statement

The study was approved by the Ethics Committee of the Valencian International University (VIU), < city>Valencia < /city>, Spain (Ref. CEID2021_07), and was conducted in accordance with the Declaration of Helsinki. Participants received information about study aims, procedures, confidentiality, potential risks, and voluntary participation, and provided written informed consent. The clinical trial registration number is NCT04813224.

## 3 Results

### 3.1 Recruitment, retention, and feasibility

**Figure 1:**
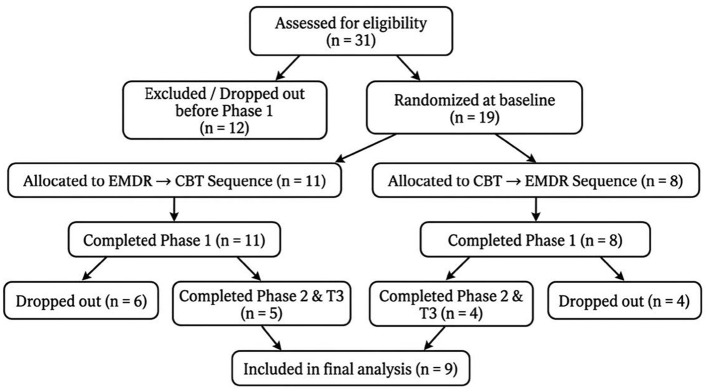
CONSORT flow diagram of participant enrollment, allocation, and retention.

Thirty-one women met eligibility criteria and entered the recruitment process. Of these, 19 completed the first 8-week treatment phase (as previously reported in Molero-Zafra et al., 2024). However, only 9 participants proceeded to complete the second 8-week crossover phase and the final T3 assessment, yielding a final retention rate of 29.0%. Therefore, the most significant attrition occurred during the transition between the first and second treatment modalities, highlighting the challenges of retaining this clinical population in extended sequential protocols. Specific individual reasons for dropout were not systematically recorded, though the timing of attrition suggests a high cumulative burden of treatment. Five completers were allocated to the EMDR → CBT sequence and four to the CBT → EMDR sequence. No serious adverse events were reported during treatment delivery. Treatment satisfaction scores (CRES-4) were generally high among completers across both treatment conditions. Given the substantial attrition observed, feasibility conclusions should be restricted to participants who remained engaged throughout the intervention. Participant flow is summarized in Figure 1.

A comprehensive summary of the primary feasibility outcomes, including retention rates, treatment parameters, and tolerability indicators, is presented in Table 1.

**Table 1:** Summary of feasibility and primary clinical outcomes.

Indicator	Result
Eligible participants	31
Completed intervention	9
Completion rate	29.0%
EMDR → CBT completers	5
CBT → EMDR completers	4
Intervention format	Online group
Number of sessions	16
Assessments	T1, T2, and T3
Treatment satisfaction (CRES-4)	High across both groups
Serious adverse events	None reported

### 3.2 Primary clinical outcomes

Within-group analyses across T1, T2, and T3 revealed descriptive changes across several clinical domains (For detailed information, see Supplementary Table S1 in the Supplementary material). Most Friedman tests did not reach statistical significance, as expected given the exploratory nature of this small pilot sample. Kendall's *W* effect sizes indicated variability in the magnitude of change across clinical outcomes and treatment sequences (For detailed information, see Supplementary Table S2 in the Supplementary material). Changes in PTSD symptoms were generally negligible to small (Kendall's *W* = 0.02–0.08), whereas larger effects were observed in specific domains, including dissociation (*W* = 0.72 in the CBT → EMDR sequence), self-esteem (*W* =0.48 in the EMDR → CBT sequence), phobic anxiety (*W* = 0.57 in the EMDR → CBT sequence), and emotional regulation strategies (*W* = 0.50 in the CBT → EMDR sequence). These findings should be interpreted as exploratory signals rather than evidence of treatment efficacy.

Effect sizes varied across outcomes and treatment sequences. In the EMDR → CBT condition, larger effects were observed for phobic anxiety, self-esteem, and emotional clarity, indicating greater changes in anxiety-related and self-related domains. Conversely, in the CBT → EMDR condition, larger effects were observed in dissociative domains, including dissociation (*W* = 0.72), absorption (*W* = 0.58), and depersonalization/derealization (*W* = 0.58). Primary clinical outcomes and corresponding effect sizes are summarized in Table 2.

**Table 2:** Primary clinical outcomes across time.

Variable	EMDR→CBT				CBT→EMDR			
	T1	T3	W	Magnitude	T1	T3	W	Magnitude
PTSD total	28.8	25.8	0.08	Small	28.5	28.5	0.02	Small
Dissociation	366	304	0.28	Small	475	287.5	0.72	Large
Self-esteem	26.8	31.6	0.48	Medium	23	22.25	0.02	Small
Life satisfaction	19.8	23.6	0.03	Small	20.25	18	0.43	Medium

Kendall's W interpretation: < 0.10 = no effect/negligible; 0.10 = small; 0.30 = moderate/medium; ≥0.50 = large. DES scores are reported as total summed scores (range 0–2,800).

Between-group comparisons indicated that most variables did not differ significantly between treatment sequences across T1, T2, or T3 (For detailed information, see Supplementary Table S3 in the Supplementary material), with the exception of isolated findings for Positive Symptomatic Distress at baseline (*p* = 0.048) and impulse-control difficulties at T3 (*p* = 0.043). Rank-biserial effect sizes revealed several medium-to-large effects despite non-significant statistical tests (For detailed information, see Supplementary Table S4 in the Supplementary material), including effects for somatization, depression, anxiety, global severity, and impulse-control difficulties.

However, due to the limited sample size (*N* = 9), the study was not powered to conduct meaningful between-group comparisons of change trajectories. Consequently, sequence-related effect sizes were not interpreted as evidence of superiority for either treatment order, but rather as preliminary indicators of potential differential patterns of symptom change associated with treatment sequencing.

### 3.3 Illustrative clinical case: TF-CBT followed by EMDR G-TEP

To illustrate the clinical trajectory suggested by the group-level findings, we summarize the course of one participant assigned to TF-CBT followed by EMDR G-TEP. The case is presented as an illustrative example only and should not be interpreted as representative of all participants.

The participant was a 30-year-old woman with complex PTSD associated with chronic intrafamilial CSA between the ages of 3 and 15 years. At baseline, she presented clinically relevant post-traumatic symptoms (EGS-R = 29), dissociative symptoms (score = 8), and global psychological distress [SCL-90-R Global Severity Index (GSI) = 0.76]. Her presentation included intrusive memories, emotional numbing, avoidance of trauma-related cues, shame, and difficulties identifying internal states during interpersonal stress. These features were clinically consistent with a complex trauma profile in which dissociation and emotion-regulation problems interfered with daily functioning and therapeutic engagement.

During the first phase, she completed eight online group TF-CBT sessions focused on psychoeducation, emotion-regulation strategies, cognitive restructuring, and trauma narrative work. Sessions emphasized recognition of trauma-related responses, identification of cognitive distortions linked to guilt and self-blame, and gradual construction of a coherent narrative. After this phase, dissociative symptoms decreased from 8 to 3, suggesting improved regulation and greater integration of bodily and cognitive experiences. However, PTSD symptoms temporarily increased, with the total EGS-R score rising from 29 to 34 and re-experiencing symptoms increasing from 4 to 6. This pattern was clinically interpreted as trauma activation during narrative and cognitive engagement rather than deterioration, as the participant simultaneously reported better understanding of her emotional responses and greater capacity to remain present during distress.

During the second phase, she completed eight online EMDR G-TEP sessions. Using the structured G-TEP worksheet, she identified several points of disturbance linked to prolonged abuse and represented them through symbolic and cognitive material, including themes of betrayal, deception, and fear. Initial subjective units of disturbance were high (SUD = 9/10), but within-session distress progressively decreased across sessions, with examples of reductions from 9 to 6 and from 4 to 2 during early processing. The participant described the process as intense but regulated emotional release, without evidence of pathological dissociation during group work. The structured protocol, therapist pacing, and repeated self-regulation steps appeared clinically important for maintaining containment in the online group setting.

At the final assessment, the total EGS-R score decreased to 27, improving on baseline and reversing the temporary increase observed after TF-CBT. Re-experiencing stabilized, and dissociative symptoms decreased further to 2. The SCL-90-R GSI stabilized at 0.88, with reductions in specific dimensions such as somatization (0.50–0.33) and stable low phobic anxiety (0.20). Although some indices did not show uniform improvement, the overall clinical picture suggested better integration and reduced trauma-related fragmentation. Qualitatively, the participant described a shift toward temporal integration of the trauma, stating that the events belonged to the past and were no longer being relived daily. She also described the combined intervention as helping her move from prolonged rumination toward a more active orientation to present life.

The trajectory observed in this illustrative participant was consistent with a possible stabilization-and-processing sequence; however, causal inferences regarding specific treatment mechanisms cannot be drawn from a single uncontrolled case. Therefore, this clinical vignette is purely illustrative and hypothesis-generating, rather than supportive evidence for a sequence-specific mechanism. Importantly, the case highlights how individual clinical changes may complement group-level quantitative analyses in very small pilot samples, without replacing the need for future controlled testing.

## 4 Discussion

The primary objective of this pilot study was to evaluate feasibility and generate preliminary clinical signals regarding two sequential online trauma-focused group interventions (TF-CBT and EMDR G-TEP) in adult women with CSA histories. A central finding was the substantial challenge of participant retention, with fewer than one-third of eligible participants completing the full protocol.

The timing of attrition provides important clinical insights. While 19 participants successfully completed the initial 8-week phase (Molero-Zafra et al., 2024), more than half of this cohort dropped out during the crossover to the second 8-week modality. This specific pattern of attrition is highly consistent with findings from Lewis et al. (2020), who demonstrated that trauma-focused PTSD interventions frequently experience substantial dropout, particularly among individuals with complex trauma presentations. In the context of our sequential design, the prospect of an additional 8-week trauma-focused protocol likely exacerbated the exact barriers identified by Lewis et al. (2020): avoidance, emotional activation, and the high cumulative burden of treatment. Furthermore, because participants already experienced initial symptom relief during Phase 1 (Molero-Zafra et al., 2024), their motivation to endure further emotional activation during a second modality may have diminished. As suggested by the literature (Lewis et al., 2020), future studies evaluating extended or sequential online protocols must prioritize engagement-enhancing procedures, such as interim support contacts, flexible scheduling, and systematic monitoring of treatment burden to mitigate this attrition.

Completion of either intervention sequence was associated with descriptive improvements across several clinical domains. Notably, while changes in overall PTSD severity were negligible to small, larger exploratory effects were observed in dissociative symptoms, particularly following the CBT → EMDR sequence. These findings are consistent with clinical models suggesting that complex trauma treatment may need to address both regulation and processing, particularly when dissociative symptoms are prominent. However, given the exploratory design and limited sample size, these findings cannot determine whether the observed pattern reflects differential treatment effects or baseline variability. The present findings extend this literature by examining a sequential format rather than a single treatment modality, and by doing so in an online group context with adult CSA survivors.

The pattern of effect sizes may provide preliminary hypotheses regarding potential differential changes across symptom domains, although these observations remain highly tentative given the sample size and baseline imbalances.

Furthermore, although the present study conceptualized TF-CBT and EMDR G-TEP as sequential treatment phases, both interventions incorporate elements of emotional regulation, stabilization, and trauma processing. Consequently, differences attributable to treatment order alone may be less pronounced than originally hypothesized. Future studies should further examine which therapeutic processes are activated within each phase and whether specific symptom profiles benefit from particular sequencing strategies.

The findings also support the potential value of online group delivery for women with CSA-related complex trauma. The G-TEP protocol embeds stabilization and self-regulation procedures, which may represent relevant components when considering tolerability in sequential formats. Group-based treatment may provide opportunities for normalization, interpersonal learning, and cohesion, which could support engagement in trauma-focused work (Yalom and Leszcz, 2020). At the same time, online delivery requires careful attention to privacy, emotional safety, crisis planning, and therapist monitoring, especially during trauma-processing sessions. In clinical practice, this means that online group EMDR should not be treated as a simple transfer of individual trauma processing to a digital setting; it requires structured preparation, clear safety procedures, and clinicians trained in both group process and trauma stabilization.

Nevertheless, the results must be interpreted primarily as feasibility and signal-detection data. The final sample was very small (*N* = 9), limiting statistical power and increasing the risk of Type II error. Effect size estimates are unstable in samples of this size, and baseline differences, including Positive Symptomatic Distress, limit direct comparison between sequences. The findings, therefore, do not establish superiority of either order; they identify patterns that may guide hypotheses for a larger trial. For a future confirmatory study, the most defensible approach would be to define one or two primary outcomes, such as dissociation and PTSD severity, and treat the remaining variables as secondary or exploratory.

Additional limitations include the non-probabilistic convenience sample, self-selection into the study, reliance on self-report measures, absence of blinded assessment, absence of long-term follow-up, and lack of correction for multiple comparisons. These factors restrict generalizability and mean that observed changes cannot be attributed confidently to the intervention or to treatment order. The online group format may also have influenced disclosure, alliance, and engagement in ways that were not directly measured. Finally, the illustrative case was selected to clarify a clinically meaningful trajectory and therefore cannot be used to estimate prevalence of response patterns within the sample.

Future studies should use larger controlled designs, preregistered primary outcomes, longer follow-up, and strategies to reduce dropout. Designs comparing shorter, stepped, or more flexible sequences may help determine whether all participants require both treatment components and which clinical profiles benefit most from each order. Future research should also evaluate mediators such as emotion regulation, dissociation, alliance, and treatment expectancy to clarify whether changes occur through distinct mechanisms across TF-CBT and EMDR G-TEP. A dismantling or adaptive design could be especially useful, as it would allow participants who respond sufficiently to the first phase to avoid unnecessary treatment burden while offering the second phase to those with persistent dissociation or trauma activation. Qualitative data could also help identify subjective indicators of readiness for trauma processing in group and online settings.

Despite these limitations, the study provides preliminary insights into the feasibility of delivering a sequential TF-CBT and EMDR G-TEP online group model among completers, while also highlighting substantial challenges related to participant retention and engagement. Its main clinical relevance lies in the possibility of organizing evidence-based interventions into a structured sequence. The results justify further research on treatment sequencing, especially for dissociative symptoms and emotion-regulation outcomes. If supported in future adequately powered studies, a sequence-informed approach could help clinicians examine whether trauma-focused interventions can be tailored according to symptom profiles while maintaining a structured and scalable format for services with limited access to specialized individual therapy.

In conclusion, both sequences were associated with clinically meaningful descriptive changes among completers, but the small sample precludes efficacy claims. The inclusion of the illustrative case suggests one possible clinical pathway; however, specific treatment mechanisms cannot be confidently disentangled from a single uncontrolled case. Treatment order may provide a relevant framework for tailoring trauma-focused interventions, and should be examined in adequately powered trials with stronger retention procedures, prespecified primary outcomes, and follow-up assessment.

## Data Availability

The original contributions presented in the study are included in the article/Supplementary material, further inquiries can be directed to the corresponding author/s.
