# Supplementary material for: A pilot study of the feasibility and preliminary outcomes of sequential TF-CBT and EMDR online group therapy for adult women with histories of childhood sexual abuse
Source: Front Psychol. 2026 Jul 15;17:1872104. doi: 10.3389/fpsyg.2026.1872104 (PMC13416442; doi:10.3389/fpsyg.2026.1872104)
Supplement: Supplementary file 1 [file Supplementary_file_1.docx]

Supplementary Material

# Table S1

| **Supplentary Table 1. Within-group differences in clinical variables across intervention times in the EMDR-CBT and CBT-EMDR groups.** | | | | | | | | | | | |
| --- | --- | --- | --- | --- | --- | --- | --- | --- | --- | --- | --- |
| Variable | EMDR-CBT Group | | | | | CBT- EMDR Group | | | | | |
|  | T1  *M (SD)* | T2  *M (SD)* | T3  *M (SD)* | *Friedman test* | *p* | T1  *M (SD)* | T2  *M (SD)* | T3  *M (SD)* | *Friedman test* | | *p* |
| **Post-traumatic Stress Disorder Symptom (EGS-R)** |  |  |  |  |  |  |  |  |  | |  |
| Re-experimentation | 5.6 (3.78) | 5.4 (3.05) | 5.2 (1.92) | .118 | .943 | 6.25 (5.19) | 5 (5.71) | 5 (4.24) | 2 | | .368 |
| Avoidance | 5.4 (3.51) | 4.4 (3.21) | 4.4 (2.61) | 3.455 | .178 | 4 (2.71) | 2.75 (2.06) | 4.5 (3.41) | 1.273 | | .529 |
| Negative alterations in cognition and mood | 11.8 (6.91) | 9 (6.12) | 10.2 (7.15) | 2.842 | .241 | 10 (6.88) | 8.5 (6.56) | 10 (6.98) | .545 | | .761 |
| Increased psychophysiological activation | 6 (5.1) | 5.6 (5.13) | 6 (4.06) | .133 | .936 | 8.25 (5.68) | 8.5 (4.79) | 9 (4.97) | 1 | | .607 |
| Dissociative symptoms | 4 (5.1) | 3.6 (3.43) | 3.4 (3.97) | .353 | .838 | 4.75 (3.3) | 4.5 (4.12) | 4.25 (3.09) | 1.4 | | .497 |
| Dysfunctionality symptoms | 7.4 (4.1) | 9.8 (6.5) | 9.2 (4.32) | .5 | .779 | 8 (6.73) | 9 (4.55) | 8.25 (4.79) | .667 | | .717 |
| Total post-traumatic stress disorder symptom (total score) | 28.8 (18.02) | 24.4 (14.79) | 25.8 (15.21) | .778 | .678 | 28.5 (20.04) | 24.75 (18.1) | 28.5 (17.86) | .133 | | .936 |
| **Psychiatric diseases (SCL-90)** |  |  |  |  |  |  |  |  |  | |  |
| Somatisations | 9.6 (8.02) | 5.6 (4.28) | 7.6 (5.37) | 1.882 | .39 | 19 (9.66) | 18.25 (12.84) | 19.5 (13.2) | .5 | | .779 |
| Obsessive-Compulsive | 13.8 (6.98) | 11.4 (7.23) | 8.6 (5.37) | 3.6 | .165 | 20.5 (8.27) | 18.25 (12.09) | 18.5 (12.29) | .143 | | .931 |
| Interpersonal sensitivity | 12.8 (9.36) | 10.2 (7.22) | 9.2 (6.06) | 2.111 | .348 | 19 (7.48) | 16.5 (10.63) | 18.75 (13.22) | 1.273 | | .529 |
| Depression | 19 (8.69) | 16.2 (10.66) | 17 (7.11) | .444 | .801 | 27.5 (8.96) | 27 (18.24) | 27.75 (18.73) | 2 | | .368 |
| Anxiety | 10.8 (7.39) | 8.8 (6.42) | 11.4 (6.11) | 1.778 | .411 | 19 (11.86) | 18.75 (12.01) | 20.5 (13.18) | 1.714 | | .424 |
| Hostility | 4 (2.45) | 4 (3.24) | 4.4 (1.67) | .778 | .678 | 7.75 (5.68) | 8.25 (8.84) | 7.75 (7.63) | .133 | | .936 |
| Phobic anxiety | 4.4 (3.21) | 1.6 (1.14) | 4.6 (5.18) | 5.692 | .058* | 7.25 (7.63) | 8 (5.71) | 9.5 (9.39) | .933 | | .627 |
| Paranoid ideation | 6.4 (3.51) | 4.2 (3.83) | 4.4 (4.09) | 3.263 | .196 | 11.25 (5.12) | 10 (6.68) | 10.25 (6.8) | .429 | .807 | |
| Psychoticism | 8.8 (8.23) | 5.8 (5.54) | 6.4 (5.37) | 2.778 | .249 | 17.75 (9.84) | 16.75 (11.18) | 17.25 (11.87) | 1.077 | | .584 |
| Additional Items | 8.2 (7.89) | 5.4 (6.58) | 5 (4.47) | .778 | .678 | 13.5 (7.23) | 12.5 (9.11) | 13 (9.31) | .133 | | .936 |
| Psychiatric disease global severity | 97.8 (60.92) | 73.2 (51.55) | 78.6 (44.79) | 2.8 | .247 | 162.5 (73.5) | 154.25 (98.97) | 162.75 (104.75) | .133 | | .936 |
| Positive Symptomatic Distress | 50.4 (18.85) | 40 (25) | 41 (20.32) | 2.8 | .247 | 74 (17.09) | 63.5 (38.73) | 62 (37.77) | .571 | | .751 |
| **Emotional Regulation Difficulties (DERS)** |  |  |  |  |  |  |  |  |  | |  |
| Lack of emotional awareness | 19.4 (5.41) | 17.4 (6.19) | 21.5 (7.42) | 1.5 | .472 | 19.25 (3.86) | 19.75 (4.5) | 16.75 (.5) | .429 | | .807 |
| Impulse control difficulties | 10.67 (7.23) | 9.4 (3.65) | 8.5 (2.08) | 2 | .368 | 15 (4.83) | 16 (6.68) | 15 (5.1) | .429 | | .807 |
| Non-acceptance of emotional responses | 16 (11.36) | 14.6 (7.83) | 15.75 (7.93) | .133 | .936 | 19.25 (8.06) | 18.75 (7.72) | 17.75 (6.65) | 1.714 | .424 | |
| Difficulties in persisting in goal-directed behaviour when emotionally aroused | 12.4 (1.67) | 11.6 (3.71) | 12.25 (4.35) | 0 | 1 | 14.75 (2.36) | 15 (2.45) | 13.75 (4.27) | .2 | | .905 |
| Lack of emotional understanding or clarity | 15.8 (7.69) | 14.2 (7.85) | 14.75 (8.34) | 3.714 | .156 | 13.75 (5.38) | 13 (5.35) | 13.25 (5.68) | 0 | | 1 |
| Limited access to emotion regulation strategies | 18.8 (8.93) | 17 (8.86) | 18.5 (9.81) | .933 | .627 | 20.5 (7) | 23 (7.35) | 23.25 (8.26) | 4 | | .135 |
| General emotional regulation difficulties (total score) | 106.33 (38.81) | 84.2 (30.38) | 91.25 (28.69) | 0 | 1 | 102.5 (28.69) | 105.5 (30.66) | 99.75 (24.13) | 1.077 | .584 | |
| **Dissociative Experiences Scale (DES)** |  |  |  |  |  |  |  |  |  | |  |
| Absorption | 108 (180.89) | 32 (34.2) | 46 (56.83) | 4.23 | .12 | 180 (148.32) | 152.5 (164.59) | 65 (58.02) | 4.667 | | .097* |
| Dissociation | 366 (283.42) | 212.5 (165.6) | 304 (179.25) | 2.8 | .247 | 475 (266.89) | 437.5 (263.99) | 287.5 (160.91) | 5.733 | | .057* |
| Depersonalization/ Derealization | 156 (197.69) | 92 (88.71) | 104 (132.21) | .778 | .678 | 167.5 (173.08) | 137.5 (178.58) | 115 (154.59) | 4.667 | | .097* |
| **Self-esteem (Rosenberg)** | 26.8 (9.2) | 30.2 (6.38) | 31.6 (6.07) | 4.778 | .092* | 23 (8.76) | 21.5 (4.51) | 22.25 (6.99) | .133 | | .936 |
| **Satisfaction with Life (SWL)** | 19.8 (7.56) | 23.2 (2.95) | 23.6 (7.7) | .333 | .846 | 20.25 (5.68) | 17.75 (6.55) | 18 (6.58) | 3.455 | | .178 |
| **Care-Receiver Efficacy (CRES-4)** |  |  |  |  |  |  |  |  |  | |  |
| Satisfaction | 73.33 (46.19) | 66.67 (41.63) | 80 (20) | 1 | .607 | 86.67 (23.09) | 73.33 (11.55) | 73.33 (11.55) | 1.4 | | .497 |
| Problem solving | 80 (34.64) | 53.33 (23.09) | 80 (0) | 2.6 | .273 | 93.33 (11.55) | 73.33 (11.55) | 86.67 (11.55) | 2.6 | | .273 |
| Perception of emotional change | 54.17 (7.22) | 58.33 (19.09) | 58.33 (7.22) | .667 | .717 | 70.83 (7.22) | 54.17 (7.22) | 58.33 (7.22) | 3.818 | | .148 |

Note: *p<0.1 **p<0.05. Trends interpreted descriptively. DES scores are reported as total summed scores (range 0–2800), rather than mean percentages

# Table S2

# Supplementary Table 2. Effect Sizes for Within-Group Longitudinal Changes

| **Scale/Variable** | **W (EMDR→CBT)** | **Magnitude** | **W (CBT→EMDR)** | **Magnitude** |
| --- | --- | --- | --- | --- |
| **Post-traumatic Stress Disorder Symptom (EGS-R)** |  |  |  |  |
| Re-experimentation | 0.01 | Small | 0.25 | Small |
| Avoidance | 0.35 | Medium | 0.16 | Small |
| Negative alterations in cognition and mood | 0.28 | Small | 0.07 | Small |
| Increased psychophysiological activation | 0.01 | Small | 0.12 | Small |
| Dissociative symptoms | 0.04 | Small | 0.17 | Small |
| Dysfunctionality symptoms | 0.05 | Small | 0.08 | Small |
| Total post-traumatic stress disorder symptom (total score) | 0.08 | Small | 0.02 | Small |
| **Psychiatric diseases (SCL-90)** |  |  |  |  |
| Somatisations | 0.19 | Small | 0.06 | Small |
| Obsessive-Compulsive | 0.36 | Medium | 0.02 | Small |
| Interpersonal sensitivity | 0.21 | Small | 0.16 | Small |
| Depression | 0.04 | Small | 0.25 | Small |
| Anxiety | 0.18 | Small | 0.21 | Small |
| Hostility | 0.08 | Small | 0.02 | Small |
| Phobic anxiety | 0.57 | Large | 0.12 | Small |
| Paranoid ideation | 0.33 | Medium | 0.05 | Small |
| Psychoticism | 0.28 | Small | 0.13 | Small |
| Additional Items | 0.08 | Small | 0.02 | Small |
| Psychiatric disease global severity | 0.28 | Small | 0.02 | Small |
| Positive Symptomatic Distress | 0.28 | Small | 0.07 | Small |
| **Emotional Regulation Difficulties (DERS)** |  |  |  |  |
| Lack of emotional awareness | 0.15 | Small | 0.05 | Small |
| Impulse control difficulties | 0.2 | Small | 0.05 | Small |
| Non-acceptance of emotional responses | 0.01 | Small | 0.21 | Small |
| Difficulties in persisting in goal-directed behaviour when emotionally aroused | 0.0 | None | 0.03 | Small |
| Lack of emotional understanding or clarity | 0.37 | Medium | 0.0 | None |
| Limited access to emotion regulation strategies | 0.09 | Small | 0.5 | Large |
| General emotional regulation difficulties (total score) | 0.0 | None | 0.13 | Small |
| **Dissociative Experience Scale (DES)** |  |  |  |  |
| Absorption | 0.42 | Medium | 0.58 | Large |
| Dissociation | 0.28 | Small | 0.72 | Large |
| Depersonalization/ Derealization | 0.08 | Small | 0.58 | Large |
| **Self-esteem (RSE)** |  |  |  |  |
| Self-esteem | 0.48 | Medium | 0.02 | Small |
| **Satisfaction with Life (SWLS)** |  |  |  |  |
| Satisfaction with Life | 0.03 | Small | 0.43 | Medium |
| **Care-Receiver Efficacy (CRES-4)** |  |  |  |  |
| Satisfaction | 0.2 | Small | 0.2 | Small |
| Problem solving | 0.15 | Small | 0.1 | Small |
| Perception of emotional change | 0.25 | Small | 0.2 | Small |

Note. Kendall’s W interpretation: .10 small, .30 medium, .50 large.

| Table S3 **Supplementary Table 3. Between-Group Differences in Clinical Variables Across Assessment Timepoints** | | | | | | | | | | | | | |
| --- | --- | --- | --- | --- | --- | --- | --- | --- | --- | --- | --- | --- | --- |
| Variable/Scale | T1 |  |  |  | T2 |  |  |  | T3 |  |  |  |  |
|  | EMDR-CBT Group  *M (SD)* | CBT- EMDR Group  *M (SD)* | *U* | *p* | EMDR-CBT Group  *M (SD)* | CBT- EMDR Group  *M (SD)* | *U* | *p* | EMDR-CBT Group  *M (SD)* | CBT- EMDR Group  *M (SD)* | *U* | *p* |  |
| **Post-traumatic Stress Disorder Symptom (EGS-R)** |  |  |  |  |  |  |  |  |  |  |  |  |  |
| Re-experimentation | 5.6 (3.78) | 6.25 (5.19) | 9 | .804 | 5.4 (3.05) | 5 (5.71) | 12.5 | .532 | 5.2 (1.92) | 5 (4.24) | 9 | .806 |  |
| Avoidance | 5.4 (3.51) | 4 (2.71) | 13.5 | .385 | 4.4 (3.21) | 2.75 (2.06) | 14 | .319 | 4.4 (2.61) | 4.5 (3.41) | 9.5 | .897 |  |
| Negative alterations in cognition and mood | 11.8 (6.91) | 10 (6.88) | 12 | .624 | 9 (6.12) | 8.5 (6.56) | 11 | .805 | 10.2 (7.15) | 10 (6.98) | 11 | .805 |  |
| Increased psychophysiological activation | 6 (5.1) | 8.25 (5.68) | 7.5 | .532 | 5.6 (5.13) | 8.5 (4.79) | 6.5 | .381 | 6 (4.06) | 9 (4.97) | 5.5 | .268 |  |
| Dissociative symptoms | 4 (5.1) | 4.75 (3.3) | 8 | .617 | 3.6 (3.43) | 4.5 (4.12) | 9 | .805 | 3.4 (3.97) | 4.25 (3.09) | 8 | .621 |  |
| Dysfunctionality symptoms | 7.4 (4.1) | 8 (6.73) | 10 | 1 | 9.8 (6.5) | 9 (4.55) | 11 | .805 | 9.2 (4.32) | 8.25 (4.79) | 12 | .624 |  |
| Total post-traumatic stress disorder symptom (total score) | 28.8 (18.02) | 28.5 (20.04) | 10 | 1 | 24.4 (14.79) | 24.75 (18.1) | 10 | 1 | 25.8 (15.21) | 28.5 (17.86) | 7 | .459 |  |
| **Psychiatric diseases (SCL-90)** |  |  |  |  |  |  |  |  |  |  |  |  |  |
| Somatisations | 9.6 (8.02) | 19 (9.66) | 4.5 | .176 | 5.6 (4.28) | 18.25 (12.84) | 4 | .14 | 7.6 (5.37) | 19.5 (13.2) | 4 | .142 |  |
| Obsessive-Compulsive | 13.8 (6.98) | 20.5 (8.27) | 19 | .142 | 11.4 (7.23) | 18.25 (12.09) | 5 | .221 | 8.6 (5.37) | 18.5 (12.29) | 5 | .219 |  |
| Interpersonal sensitivity | 12.8 (9.36) | 19 (7.48) | 6 | .327 | 10.2 (7.22) | 16.5 (10.63) | 4 | .142 | 9.2 (6.06) | 18.75 (13.22) | 4.5 | .176 |  |
| Depression | 19 (8.69) | 27.5 (8.96) | 4.5 | .176 | 16.2 (10.66) | 27 (18.24) | 5 | .221 | 17 (7.11) | 27.75 (18.73) | 5 | .221 |  |
| Anxiety | 10.8 (7.39) | 19 (11.86) | 5 | .219 | 8.8 (6.42) | 18.75 (12.01) | 4 | .14 | 11.4 (6.11) | 20.5 (13.18) | 4.5 | .176 |  |
| Hostility | 4 (2.45) | 7.75 (5.68) | 6.5 | .385 | 4 (3.24) | 8.25 (8.84) | 9 | .802 | 4.4 (1.67) | 7.75 (7.63) | 9.5 | .901 |  |
| Phobic anxiety | 4.4 (3.21) | 7.25 (7.63) | 10 | 1 | 1.6 (1.14) | 8 (5.71) | 3.5 | .108 | 4.6 (5.18) | 9.5 (9.39) | 7 | .457 |  |
| Paranoid ideation | 6.4 (3.51) | 11.25 (5.12) | 4.5 | .176 | 4.2 (3.83) | 10 (6.68) | 4.5 | .174 | 4.4 (4.09) | 10.25 (6.8) | 5 | .213 |  |
| Psychoticism | 8.8 (8.23) | 17.75 (9.84) | 4.5 | .174 | 5.8 (5.54) | 16.75 (11.18) | 4 | .132 | 6.4 (5.37) | 17.25 (11.87) | 5 | .219 |  |
| Additional Items | 8.2 (7.89) | 13.5 (7.23) | 6 | .323 | 5.4 (6.58) | 12.5 (9.11) | 5.5 | .266 | 5 (4.47) | 13 (9.31) | 4.5 | .176 |  |
| Psychiatric disease global severity | 97.8 (60.92) | 162.5 (73.5) | 4 | .142 | 73.2 (51.55) | 154.25 (98.97) | 4 | .14 | 78.6 (44.79) | 162.75 (104.75) | 5 | .221 |  |
| Positive Symptomatic Distress | 50.4 (18.85) | 74 (17.09) | 2 | .048** | 40 (25) | 63.5 (38.73) | 4 | .142 | 41 (20.32) | 62 (37.77) | 5 | .221 |  |
| **Emotional Regulation Difficulties (DERS)** |  |  |  |  |  |  |  |  |  |  |  |  |  |
| Lack of emotional awareness | 19.4 (5.41) | 19.25 (3.86) | 10 | 1 | 17.4 (6.19) | 19.75 (4.5) | 6 | .323 | 21.5 (7.42) | 16.75 (.5) | 12 | .237 |  |
| Impulse control difficulties | 10.67 (7.23) | 15 (4.83) | 3 | .289 | 9.4 (3.65) | 16 (6.68) | 3 | .086* | 8.5 (2.08) | 15 (5.1) | 1 | .043** |  |
| Non-acceptance of emotional responses | 16 (11.36) | 19.25 (8.06) | 7 | .462 | 14.6 (7.83) | 18.75 (7.72) | 7 | .459 | 15.75 (7.93) | 17.75 (6.65) | 7 | .772 |  |
| Difficulties in persisting in goal-directed behaviour when emotionally aroused | 12.4 (1.67) | 14.75 (2.36) | 3.5 | .102 | 11.6 (3.71) | 15 (2.45) | 4 | .14 | 12.25 (4.35) | 13.75 (4.27) | 6 | .564 |  |
| Lack of emotional understanding or clarity | 15.8 (7.69) | 13.75 (5.38) | 11.5 | .711 | 14.2 (7.85) | 13 (5.35) | 11 | .806 | 14.75 (8.34) | 13.25 (5.68) | 9.5 | .661 |  |
| Limited access to emotion regulation strategies | 18.8 (8.93) | 20.5 (7) | 8 | .624 | 17 (8.86) | 23 (7.35) | 4.5 | .176 | 18.5 (9.81) | 23.25 (8.26) | 6 | .559 |  |
| General emotional regulation difficulties (total score) | 106.33 (38.81) | 102.5 (28.69) | 7 | .724 | 84.2 (30.38) | 105.5 (30.66) | 7.5 | .539 | 91.25 (28.69) | 99.75 (24.13) | 8.5 | .884 |  |
| **Dissociative Experience Scale (DES)** |  |  |  |  |  |  |  |  |  |  |  |  |  |
| Absorption | 108 (180.89) | 180 (148.32) | 7 | .455 | 32 (34.2) | 152.5 (164.59) | 3 | .085* | 46 (56.83) | 65 (58.02) | 6.5 | .381 |  |
| Dissociation | 366 (283.42) | 475 (266.89) | 7 | .462 | 212.5 (165.6) | 437.5 (263.99) | 3 | .149 | 304 (179.25) | 287.5 (160.91) | 12.5 | .539 |  |
| Depersonalization/ Derealization | 156 (197.69) | 167.5 (173.08) | 9 | .805 | 92 (88.71) | 137.5 (178.58) | 9 | .806 | 104 (132.21) | 115 (154.59) | 9.5 | .902 |  |
| **Self-esteem (Rosenberg)** | 26.8 (9.2) | 23 (8.76) | 12 | .621 | 30.2 (6.38) | 21.5 (4.51) | 18 | .049** | 31.6 (6.07) | 22.25 (6.99) | 17 | .086* |  |
| **Satisfaction with Life (SWL)** | 19.8 (7.56) | 20.25 (5.68) | 8 | .623 | 23.2 (2.95) | 17.75 (6.55) | 16 | .14 | 23.6 (7.7) | 18 (6.58) | 15 | .221 |  |
| **Care-Receiver Efficacy (CRE)** |  |  |  |  |  |  |  |  |  |  |  |  |  |
| Satisfaction | 73.33 (46.19) | 86.67 (23.09) | 4 | .796 | 66.67 (41.63) | 73.33 (11.55) | 7.5 | 1 | 80 (20) | 73.33 (11.55) | 5.5 | .637 |  |
| Problem solving | 80 (34.64) | 93.33 (11.55) | 4 | .796 | 53.33 (23.09) | 73.33 (11.55) | 4.5 | .334 | 80 (0) | 86.67 (11.55) | 3 | .317 |  |
| Perception of emotional change | 54.17 (7.22) | 70.83 (7.22) | .5 | .068* | 58.33 (19.09) | 54.17 (7.22) | 7.5 | 1 | 58.33 (7.22) | 58.33 (7.22) | 4.5 | 1 |  |
| Note: *p<0.1 **p<0.05. Trends interpreted descriptively. DES scores are reported as total summed scores (range 0–2800), rather than mean percentages | | | | | | | | | | | | | |

# Table S4

# Supplementary Table 4. Effect Sizes (r_rb) for Between-Group Comparisons

| Scale/Variable | T1 r_rb | Mag | T2 r_rb | Mag | T3 r_rb | Mag |
| --- | --- | --- | --- | --- | --- | --- |
| **EGS-R** |  |  |  |  |  |  |
| Re-experimentation | 0.1 | Small | -0.25 | Small | 0.1 | Small |
| Avoidance | -0.35 | Medium | -0.4 | Medium | 0.05 | Small |
| Negative alterations in cognition and mood | -0.2 | Small | -0.1 | Small | -0.1 | Small |
| Increased psychophysiological activation | 0.25 | Small | 0.35 | Medium | 0.45 | Medium |
| Dissociative symptoms | 0.2 | Small | 0.1 | Small | 0.2 | Small |
| Dysfunctionality symptoms | 0.0 | None | -0.1 | Small | -0.2 | Small |
| Total PTSD | 0.0 | None | 0.0 | None | 0.3 | Medium |
| **SCL-90** |  |  |  |  |  |  |
| Somatisations | 0.55 | Large | 0.6 | Large | 0.6 | Large |
| Obsessive-Compulsive | -0.9 | Large | 0.5 | Large | 0.5 | Large |
| Interpersonal sensitivity | 0.4 | Medium | 0.6 | Large | 0.55 | Large |
| Depression | 0.55 | Large | 0.5 | Large | 0.5 | Large |
| Anxiety | 0.5 | Large | 0.6 | Large | 0.55 | Large |
| Hostility | 0.35 | Medium | 0.1 | Small | 0.05 | Small |
| Phobic anxiety | 0.0 | None | 0.65 | Large | 0.3 | Medium |
| Paranoid ideation | 0.55 | Large | 0.55 | Large | 0.5 | Large |
| Psychoticism | 0.55 | Large | 0.6 | Large | 0.5 | Large |
| Additional Items | 0.4 | Medium | 0.45 | Medium | 0.55 | Large |
| Psychiatric global severity | 0.6 | Large | 0.6 | Large | 0.5 | Large |
| Positive Symptomatic Distress | 0.8 | Large | 0.6 | Large | 0.5 | Large |
| **DERS** |  |  |  |  |  |  |
| Lack of emotional awareness | 0.0 | None | 0.4 | Medium | -0.2 | Small |
| Impulse control difficulties | 0.7 | Large | 0.7 | Large | 0.9 | Large |
| Non-acceptance | 0.3 | Medium | 0.3 | Medium | 0.3 | Medium |
| Goal-directed behaviour | 0.65 | Large | 0.6 | Large | 0.4 | Medium |
| Emotional clarity | -0.15 | Small | -0.1 | Small | 0.05 | Small |
| Limited strategies | 0.2 | Small | 0.55 | Large | 0.4 | Medium |
| DERS total | 0.3 | Medium | 0.25 | Small | 0.15 | Small |
| Absorption | 0.3 | Medium | 0.7 | Large | 0.35 | Medium |
| **DES** |  |  |  |  |  |  |
| Dissociation | 0.3 | Medium | 0.7 | Large | 0.25 | Small |
| Depersonalization | 0.1 | Small | 0.1 | Small | 0.05 | Small |
| **Self-esteem** | -0.2 | Small | -0.8 | Large | -0.7 | Large |
| **Life satisfaction** | 0.2 | Small | -0.6 | Large | -0.5 | Large |
| **CRES-4** |  |  |  |  |  |  |
| Satisfaction | 0.6 | Large | 0.25 | Small | 0.45 | Medium |
| Problem solving | 0.6 | Large | 0.55 | Large | 0.7 | Large |
| Emotional change | 0.95 | Large | 0.25 | Small | 0.55 | Large |

Note. r_rb = 1 − (2U / (n1 × n2) n1=5, n2=4. Interpretation: .10 small, .30 medium, .50 large.
